# Changes in Fish Taxonomic and Phylogenetic Diversity and Their Driving Factors in a Reservoir in the Karst Basin of Southwest China

**DOI:** 10.3390/ani16010145

**Published:** 2026-01-05

**Authors:** Jialing Qiao, Yang Liu, Weiwei Yao, Hong Ma, Liang Yu, Qin Zhao, Lijian Ouyang

**Affiliations:** 1 College of Ecological Engineering, Guizhou University of Engineering Science, Bijie 551700, China; qiaojialing9406@126.com (J.Q.); yangliuouc1102@163.com (Y.L.); 19336672767@163.com (H.M.); mynamesyuliang1016@163.com (L.Y.); 18212807432@163.com (Q.Z.); 2 Institute of Fishery Science, Hangzhou Academy of Agricultural Sciences, Hangzhou 310024, China; 3 State Key Laboratory of Hydraulics and Mountain River Engineering, Sichuan University, Chengdu 610065, China; yaoww@scu.edu.cn

**Keywords:** lentic ecosystem, multidimensional diversity, alpha and beta diversity, environmental heterogeneity, geographical distance, community assembly

## Abstract

Dam construction can significantly alter local habitat characteristics and the distribution patterns of aquatic organisms. However, the variations in the multidimensional diversity of fish assemblages in reservoirs, as well as the relative significance of potential community assembly rules, remains poorly understood. This study elucidated the patterns of taxonomic and phylogenetic α- and β-diversity with its decomposition components (i.e., turnover and nestedness) of fish assemblages in the Dongfeng Reservoir, situated in the karst basin of southwest China. Additionally, we evaluated the relative importance of environmental heterogeneity and spatial structure. We found significant nonlinear relationships (*p* < 0.05) between taxonomic and phylogenetic richness. Both fish taxonomic and phylogenetic β-diversity values were low (<0.33) with high turnover patterns (72.23% and 67.42%), underscoring the necessity for local managers to protect entire water areas to maintain or enhance community diversity. Only taxonomic and phylogenetic richness are significantly positively (e.g., water depth) and negatively (e.g., turbidity) affected by different environmental variables. Environmental heterogeneity was the dominant factor influencing both total β-diversity and turnover processes at the taxonomic and phylogenetic levels, while spatial distance primarily influenced the nestedness process. These findings are critical for elucidating changes in patterns of fish community diversity and their driving factors in the context of dam construction, providing a foundation for the conservation and management of aquatic organisms in other rivers, lakes, and reservoirs.

## Simple Summary

We examined changes in the environmental characteristics of reservoir impoundment and analyzed patterns of fish taxonomic and phylogenetic α- and β-diversity. A few significant relationships between taxonomic and phylogenetic α-diversity indicated that considering only the diversity of a single dimension might lead to deviations in results and conclusions. The formation of reservoir impoundment increased taxonomic and phylogenetic similarity. The importance of community assembly mechanisms varied based on the ecological processes measured; total β-diversity and the turnover process for taxonomic and phylogenetic indices were primarily influenced by environmental heterogeneity, while the nestedness process was predominantly affected by spatial structure. Our findings highlight that dam construction alters the relationships between taxonomic and phylogenetic α-diversity from different perspectives and enhances community similarity, thereby providing a foundation for managing hydraulic infrastructure projects and conserving biodiversity.

## 1. Introduction

In comparison to marine and terrestrial ecosystems, freshwater biodiversity faces significant threats from a variety of factors (e.g., dam construction) and shows a more pronounced declining trend [1,2]. The management and protection of freshwater environments and biodiversity have long been significantly underestimated, and it is only in recent years that they have gradually attracted the attention of managers and ecologists [3,4,5]. Revealing the distribution patterns of community organisms and their formation mechanisms is essential for improving biodiversity conservation and management, as well as for sustaining ecosystem functions and services. This topic is of significant interest in fields such as community ecology and biogeography [6,7,8,9].

Previous studies on biodiversity responses to disturbance gradients primarily focused on taxonomic-level assessments, emphasizing species abundance and distribution patterns across specific spatial scales (α-diversity) and variations in species composition between communities (β-diversity) [10,11,12]. The total β-diversity can be partitioned into two additive components: species turnover and nestedness that arise from differences in species richness [11]. Species turnover encapsulates the simultaneous processes of species loss and gain between communities, potentially influenced by environmental filtering, biological interactions, and historical factors; species nestedness reflects communities with fewer species are subsets of those with greater species richness, which may arise from dispersal barriers and the underlying mechanisms that influence colonization and extinction [13,14]. Decomposing β-diversity into distinct ecological processes and linking these processes to the drivers of metacommunity structures offers deeper insights into the mechanisms underlying community diversity patterns across various spatio-temporal scales, thereby informing conservation strategies [15,16,17]. For example, high species turnover rates suggest that protecting entire communities is essential for safeguarding those with numerous distinct species, while increased nestedness implies that conserving species-rich sites can help maintain higher diversity [7].

In comparison to the taxonomic identity of species, phylogenetic information enhances our understanding of the structure and function of community organisms, as well as the ecological and evolutionary mechanisms [18,19,20,21]. Phylogenetic diversity serves as a robust proxy for both species identity and trait diversity within biological communities [22,23]. Analogously, phylogenetic β-diversity can be subdivided into phylogenetic turnover (i.e., referring to changes in evolutionary relationships of species between communities) and phylogenetic diversity gradients (i.e., describing the extent to which community with shorter phylogenetic length are subsets of those with longer relationships) [24]. Integrating species taxonomic and phylogenetic information aids in elucidating the response of community biogeographic patterns to disturbance factors and their driving mechanisms from different perspectives [25].

Niche and neutral theories are critical factors effecting species distribution and shaping community structure. The former emphasizes deterministic processes, such as environmental filtering and biological interactions, that lead to interspecific clustering and dispersion phenomena; in contrast, the latter highlights stochastic processes, where different species exhibit identical rates of formation and dispersal (e.g., dispersal limitations) [26,27]. Environmental filtering explains why certain species aggregate in suitable habitats due to environmental screening, while dispersal limitation denotes how geographical barriers or variations in dispersal capacity hinder some species from spreading to other areas [28]. Beta diversity patterns are primarily influenced by environmental heterogeneity and the geographic distance between sites, typically increasing with greater environmental variations and geographic isolation [29,30]. By utilizing environmental difference and geographic distances to model distance decay relationships of organisms between communities, researchers can elucidate the importance and relative roles of niche and neutral processes in β-diversity [29]. However, the response of community turnover and nestedness patterns of the metacommunity to environmental characteristics and spatial structure remains unclear [31]. For example, the turnover process of fish communities in the three cascade reservoirs in Brazil was mainly significantly affected by reservoir area, depth, water residence time and nitrate, while the nestedness process was only affected by the reservoir area [32].

Dam construction can regulate a river’s natural runoff and seasonal variations, serving essential functions such as hydropower generation and water supply (https://www.icold-cigb.org/, accessed on 18 October 2025). However, dam construction reduces both longitudinal and lateral connectivity, resulting in river fragmentation, which transforms flowing river segments into static or slow-flowing status, altering environmental characteristics such as sedimentation and water temperature in the reservoir. Consequently, these changes affect the distribution dynamics of communities and the structure of food webs for aquatic organisms [33,34]. For instance, as the duration of reservoir impoundment increases, overall fish species richness tends to decline. When differentiating species types, this decline is characterized by a reduction in the richness of native species and an increase in the richness of non-native species, which further increasing community dissimilarity [35]. The karst region in southwest China has a complex topography and high environmental heterogeneity, which makes aquatic organisms like fish in the watershed easily affected by dam construction. Nevertheless, few studies have investigated how taxonomic and phylogenetic diversity of fish assemblages respond to the consequences of reservoirs [36,37], especially in karst watersheds.

This study investigates the Dongfeng Reservoir, which has been established for three decades in the karst basin of southwest China, as a case study. We analyze the responses of fish taxonomic and phylogenetic α- and β-diversity with its decomposition components (i.e., turnover and nestedness) to dam construction, and explore the mechanisms underlying community assembly. Specifically, we first examined the nonlinear relationships between taxonomic and phylogenetic α-diversity of fish assemblages using generalized additive models, and assessed the relationships between β-diversity and its additive components through generalized linear models. We hypothesize that there is a significant correspondence between fish taxonomic and phylogenetic β-diversity, with greater variability in species composition at the taxonomic level [30]. Furthermore, we employed correlation analysis to identify key environmental variables impacting fish taxonomic and phylogenetic α-diversity, and integrated the distance-decay method with hierarchical partitioning analysis to evaluate the relative importance of environmental heterogeneity and spatial distance on different dimensions of β-diversity. Dam construction is expected to reduce differences in environmental conditions within the impoundment area characterized by high connectivity [38]. We anticipate that the effect of environmental heterogeneity on fish taxonomic and phylogenetic β-diversity with its decomposition components will be less pronounced than that of spatial distance.

## 2. Materials and Methods

### 2.1. Study Area

**Figure 1:**
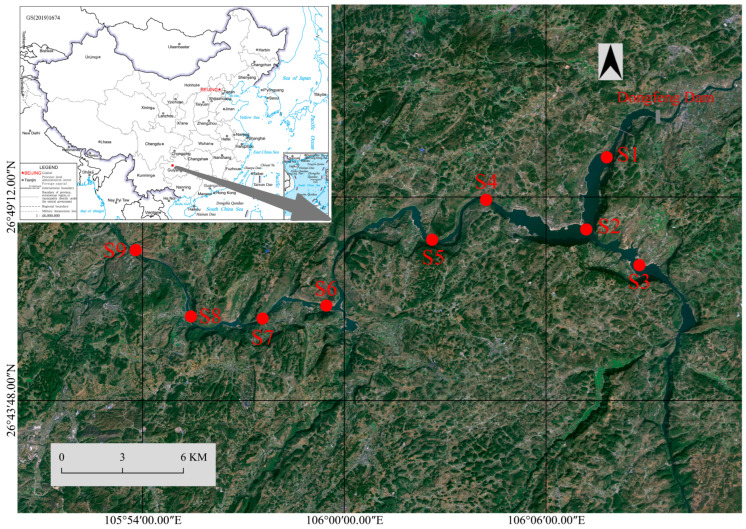
The map of field survey in the Dongfeng Reservoir. Red points represent the sampling sites.

The Dongfeng Reservoir (dam site: 106°09′ E, 26°51′ N) is situated in the northwest of Guizhou province. It encompasses a large and fork-shaped lake formed by the confluence of the Liuchong River and Sancha River, both of which are located in the upper reaches of the Wujiang River in the Yangtze River watershed (Figure 1). This reservoir exemplifies a typical karst high-mountain gorge topography, with a regional average elevation exceeding 1200 m. Its catchment area is 18,161 km^2^ and is characterized by an average annual flow of 345 m^3^/s and a water retention time of 28 days [39]. Situated within a subtropical monsoon climate zone, the reservoir area benefits from ample water resources, primarily sustained by annual precipitation that exceeds 1000 mm [40]. The total storage capacity of the reservoir is 10.25 × 10^8^ m^3^, with a normal storage level of 970 m with a capacity of 8.64 × 10^8^ m^3^ and a dead storage level of 936 m with a capacity of 3.73 × 10^8^ m^3^ (https://dam.nea.gov.cn/, accessed on 18 October 2025).

### 2.2. Field Survey

In November 2024, a total of nine sampling sites were established in the Dongfeng Reservoir, organized by their distance from the dam (Figure 1). Each site was situated in open water with a minimum depth of 2 m, thereby reducing potential impacts from varying habitat types [41]. Two cage nets (mesh size: 1 cm; length: 50 m; width: 0.3 m; height: 0.5 m) were deployed along both banks of the sampled water area, accompanied by gillnets of varying specifications in the afternoon. Specifically, there are two gillnets with a mesh size of 4 cm, two gillnets with a mesh size of 5 cm, and one gillnet with a mesh size of 12 cm. Each gillnet measures 100 m in length and 1 m in width. The nets were retrieved the following morning to collect catches from different water layers after at least 12 h. The use of multiple fishing gear types in the field allowed for a more comprehensive assessment of fish composition [42]. On-site identification and counting of the catch were conducted to the species level, utilizing references such as the “Fish Fauna of Guizhou” [43] and “Fishes of Guizhou” [44]. After biological measurements, live catches were returned to the water. Fish specimens were preserved in 8% formaldehyde and transported to the laboratory for further analysis. Species classification references included *FishBase* (www.fishbase.org, accessed on 18 October 2025) and *Catalog of Fishes* (https://www.calacademy.org/scientists/projects/eschmeyers-catalog-of-fishes, accessed on 18 October 2025). The species accumulation curve was analyzed to evaluate the sampling completeness of individual site (94.25% to 99.24%) as well as the overall sampling completeness (99.67%) and effectiveness across all sampling locations (Figure S1) using the “iNEXT” package in R version 4.2.2 [45].

At each sampling site, three cross-sections were surveyed, with three sampling points recorded per cross-section to acquire mean values for various environmental parameters. In situ measurements included water temperature (WT, °C) and pH, obtained using a portable water quality analyzer (YSI Professional Plus, Yellow Springs, OH, USA). Water depth (WD, m) and turbidity (NTU) were determined using a depth sounder and turbidity meter, respectively. Mixed water samples of 2 L were collected on-site using an acrylic water sampler and subsequently transported to the laboratory under low-temperature conditions. The water quality analysis adhered to the guidelines established by the technical regulation for the preservation and handling of samples (HJ 493-2009) [46] and encompassed the measurement of parameters such as permanganate index (COD_Mn_, mg/L), chemical oxygen demand (COD, mg/L), total phosphorus (TP, mg/L), total nitrogen (TN, mg/L), sulfate (SO_4_^2−^, mg/L), nitrite nitrogen (NN, mg/L), and chlorophyll a (CHL.a, μg/mL).

### 2.3. Taxonomic Diversity

Integrating multidimensional indices enables a comprehensive analysis of the relationships among community diversity, environmental factors, and ecosystem functions [21]. In this study, the Margalef index and the Pielou index are employed to represent community taxonomic richness and evenness, respectively. The community data undergo Hellinger transformation, which quantifies the dispersion of each sample point relative to the community centroid, reflecting community divergence.

We computed paired taxonomic β-diversity based on the Sørensen dissimilarity index using species presence and absence data. A value of 1 signifies no shared species between communities, while a value of 0 signifies complete overlap of species. Furthermore, the total β-diversity can be deconstructed into two distinct components: species turnover and nestedness, each ranging from 0 to 1. Community β-diversity metrics were computed using the “betapart” package in R version 4.2.2 [47].

### 2.4. Phylogenetic Diversity

This study utilized publicly available data from GenBank (https://www.ncbi.nlm.nih.gov/genbank/ (accessed on 18 October 2025)) to acquire mitochondrial genes (cytb and COI) of fish, aiming to establish the evolutionary relationships among species (Figure S2). We selected mitochondrial genes of fish in waters close to the investigation area to mitigate incorrect phylogenetic relationships. The GenBank code for each species was shown in Table S1. The sequences of both mitochondrial genes were aligned using MAFFT version 7.520. Subsequently, trimAl version 1.2 and Mesquite version 3.70 were employed to trim the aligned sequences and compile the sequences [48,49]. Additionally, the maximum-likelihood method and Smart Model Selection in PhyML version 3.0 were applied, performing 1000 bootstrap replicates [50,51]. Based on the criterion of the lowest Akaike Information Criterion (AIC) value, we selected the “GTR + R” model. A ultrametric tree for the reservoir fish was constructed by randomly modifying polymorphisms on the phylogenetic tree using the “ape” package in R version 4.2.2 [52,53].

We integrated a constructed phylogenetic tree with fish community composition data to assess phylogenetic richness, divergence, and evenness through Faith’s phylogenetic diversity (PD), abundance-based mean pairwise distances (MPD.abu), and abundance-based variation of pairwise distances (VPD.abu) [21]. This method enabled a comparative analysis of community α-diversity at both taxonomic and phylogenetic levels. Specifically, PD represents the cumulative branch lengths of species in the phylogenetic tree, while MPD.abu and VPD.abu denote the mean and variance of phylogenetic distances among all species in the community, weighted by abundance, respectively. To reduce the impact of species richness, we employed the “taxa.labels” model, which retains the original species order in the community matrix, and randomly permuted the terminal species’ positions of tree 999 times to calculate the standardized effect size (SES) of randomized phylogenetic α-diversity. The SES formula is defined as (Obs.phy.alpha − Mean.rand.phy.alpha)/SD.rand.phy.alpha, where Obs.phy.alpha indicates the observed phylogenetic α-diversity, and Mean.rand.phy.alpha and SD.rand.phy.alpha denote the mean and standard deviation of the index following randomization, respectively. Calculations for phylogenetic α-diversity and the corresponding SES values were performed using the “pez” and “picante” packages in R version 4.2.2 [54,55].

Phylogenetic β-diversity measures the similarity in evolutionary branch lengths between endemic and shared species between communities by employing the Sorensen dissimilarity index. When species in disparate communities are distantly related, this index nears its maximum value of 1; conversely, it approaches its minimum value of 0. Phylogenetic β-diversity can be mathematically categorized into two patterns: phylogenetic turnover and phylogenetic diversity gradients [24,47]. The former signifies the replacement of species branch lengths between communities within the phylogenetic tree, while the latter illustrates nested evolutionary relationships among species between communities. Moreover, we draw on existing computational methods to quantify the deviation pattern between taxonomic and phylogenetic total β-diversity, represented by the formula beta.dev = (βtax − βphy)/βtax. In this equation, beta.dev reflects the relationship between taxonomic (βtax) and phylogenetic β-diversity (βphy) [30,56]. Positive values indicate that communities have many endemics with close phylogenetic relationships, suggesting that taxonomic β-diversity surpasses phylogenetic diversity. In contrast, negative values denote communities shared by numerous distantly related species, indicating that taxonomic β-diversity is lower than phylogenetic diversity. When communities consist solely of distantly related endemics or closely related shared species, the value of beta.dev approaches zero.

### 2.5. Statistical Analysis

To investigate the impact of dam construction on the water environmental characteristics of local habitats, we employed generalized additive models (GAMs) to analyze the nonlinear variations of environmental variables and environmental heterogeneity (dependent variables) across sampling points at varying distances from the dam location (independent variable). Environmental heterogeneity is quantified by the mean Euclidean distance of each standardized environmental factor to the group center, with a higher mean distance indicating greater environmental heterogeneity. The GAMs utilize the generalized cross-validation (GCV) criterion to select the smoothing parameter function, providing the adjusted R^2^, significance, and deviance explained (DE) for each model [57]. Prior to analysis, the Shapiro–Wilk test was conducted to assess the normality of each environmental variable [58], with those found to be non-normally distributed undergoing log transformation. Additionally, the Gaussian distribution was specified as the “family” function. The GAM model was implemented using the “mgcv” package and the “gam” function in R version 4.2.2 [57].

The dominance of species is assessed using the relative importance index (*IRI*), defined as *IRI* = 10,000 × F*i* × (N*i* + W*i*), where F*i* represents the occurrence frequency of species *i*, N*i* denotes the proportion of individuals, and W*i* signifies the proportion of biomass for species *i*. The values of *IRI* exceeding 500 are classified as dominant species, those with values between 100 and 500 as common species, and those below 100 as rare species [59].

We employed GAMs to analyze the nonlinear relationships among taxonomic and phylogenetic richness, divergence, and evenness, and their SES values, aiming to explore potential associations between taxonomic (independent variable) and phylogenetic α-diversity (dependent variable) of fish assemblage within the watershed. To evaluate inter-community diversity, we utilized generalized linear models to examine the fitting trends of total β-diversity and its decomposed components at both taxonomic and phylogenetic levels. Subsequently, Tukey’s HSD test (α = 0.05) was performed for comparative analysis to identify significant differences in inter-community taxonomic and phylogenetic β-diversity. We used Cohen’s d to represent the effect size of taxonomic and phylogenetic β-diversity to complement the results of the significance *p* value. The analyses were conducted using the ‘glht’ function from the “multcomp” package in R version 4.2.2 [60].

To investigate the factors influencing fish taxonomic and phylogenetic α-diversity, we utilized Spearman correlation analysis to identify significant positive and negative correlations between different α-diversity indices and environmental characteristics, as well as spatial distance (i.e., the distance from each sampling site to the dam location). The Benjamini-Hochberg method was performed for adjusting the significance. For community β-diversity, a distance-decay method was employed to examine the effects of environmental heterogeneity and spatial structure (i.e., river distances between sampling points) on taxonomic and phylogenetic β-diversity and its decomposed components [29]. The distances from the sampling points to the dam location and between sampling points were calculated using ArcGIS 10.8.1 software. Additionally, hierarchical partitioning analysis was conducted to quantify the relative importance of environmental heterogeneity and spatial structure using the “glmm.hp” package in R version 4.2.2 [61], which complements the results of the distance-decay analysis. This method computes the unique marginal R^2^ for each fixed effect through ‘average shared variance’ in multivariate analysis, thus allowing for a comparison of the relative importance of different explanatory variables.

## 3. Results

### 3.1. The Distribution Patterns of Environmental Characteristics of the Dongfeng Reservoir

**Figure 2:**
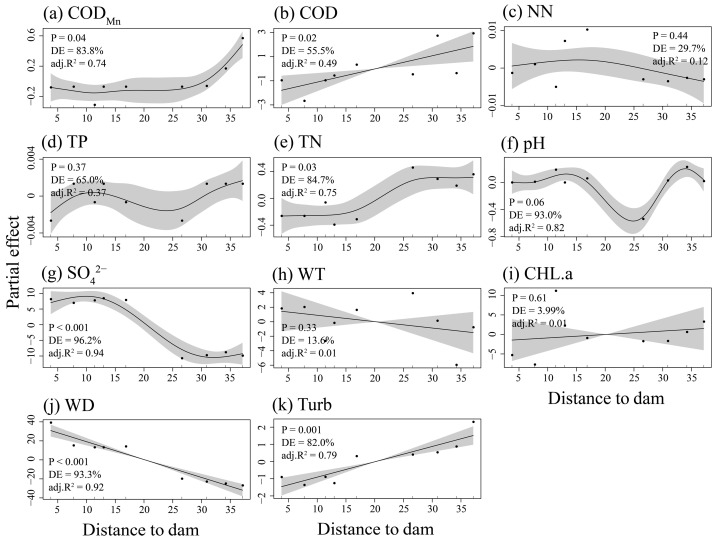
Changes in water environmental characteristics of the Dongfeng Reservoir with distance from the dam. The shaded areas indicate the 95% confidence intervals of the fitted curves.

The results showed that nearly half of the environmental variables vary with distance from the dam location (Figure 2). Specifically, COD_Mn_ (adj.R^2^ = 0.74, *p* = 0.04, Deviance explained (DE) = 83.8%), COD (adj.R^2^ = 0.49, *p* = 0.02, DE = 55.5%), TN (adj.R^2^ = 0.75, *p* = 0.03, DE = 84.7%), and turbidity (adj.R^2^ = 0.79, *p* < 0.001, DE = 82%) exhibited significant increases, while SO_4_^2−^ (adj.R^2^ = 0.94, *p* < 0.001, DE = 96.2%) and WD (adj.R^2^ = 0.92, *p* < 0.001, DE = 93.3%) demonstrated decreasing trends. Among these variables, COD_Mn_, TN, and SO_4_^2−^ represented nonlinear trends. Conversely, other environmental factors showed no significant sensitivity to distance from the dam (*p* > 0.05). Furthermore, although environmental heterogeneity at each site increased with distance from the dam, the relationship was not statistically significant (*p* = 0.22, Figure S3).

### 3.2. Fish Composition in the Dongfeng Reservoir

A total of 23 fish species from 10 families and 5 orders were surveyed in the Dongfeng Lake (Table 1). The average number of species, abundance, and biomass per site were 11.11 species, 135.00 individuals, and 7.87 kg, respectively. The results showed that the dominant species included Sharpbelly (*Hemiculter leucisculus*, *IRI* = 863.69), Goldfish (*Carassius auratus*, *IRI* = 3724.24), *Pseudogyrinocheilus prochilus* (*IRI* = 721.86), Redbelly tilapia (*Coptodon zillii*, *IRI* = 3711.27), and Green sunfish (*Lepomis cyanellus*, *IRI* = 6006.23). Furthermore, the invasive species *C. zillii* and *L. cyanellus* were caught at all sampling sites.

### 3.3. The Relationships Between Fish Taxonomic and Phylogenetic Diversity in the Dongfeng Reservoir

**Figure 3:**
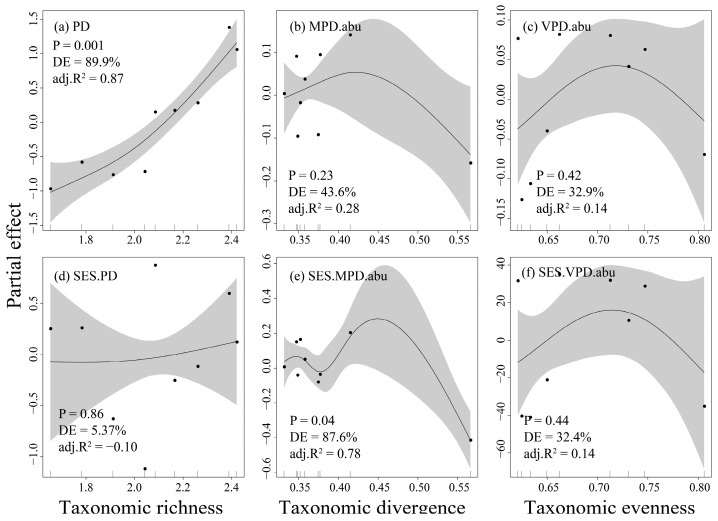
Nonlinear relationships between fish taxonomic and phylogenetic richness (**a**,**d**), divergence (**b**,**e**), and evenness (**c**,**f**) in the Dongfeng Reservoir. The shaded areas indicate the 95% confidence intervals for the fitted curves.

In terms of community α-diversity, only the phylogenetic richness demonstrated a significant increase corresponding to rising taxonomic richness (adj.R^2^ = 0.87, *p* = 0.001, DE = 89.9%, Figure 3a). When controlling for species richness, fish assemblages exhibited only irregular significance between taxonomic and randomized phylogenetic divergence (adj.R^2^ = 0.78, *p* = 0.04, DE = 87.6%, Figure 3e). However, no significant relationships were observed between taxonomic and phylogenetic evenness (*p* > 0.05, Figure 3c,f).

**Figure 4:**
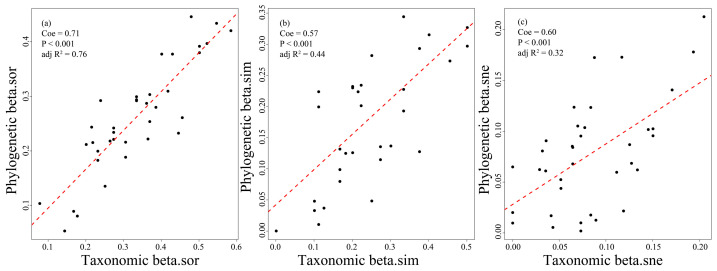
Fitting relationships between total β-diversity (**a**) and its decomposition components ((**b**) for turnover and (**c**) for nestedness) in the fish assemblages of the Dongfeng Reservoir. The red dashed line indicates the fitted curve. Coe is the fitted coefficient.

**Figure 5:**
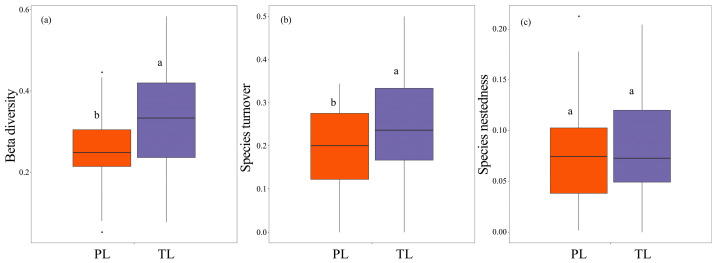
The differences between taxonomic and phylogenetic total β-diversity (**a**) and its additive components ((**b**) for species turnover and (**c**) for species nestedness) of fish assemblages in the Dongfeng reservoir. Different lowercase letters represent significant differences among groups (*p* < 0.05). TL and PL are taxonomic level and phylogenetic level, respectively.

The results revealed a significant relationship between taxonomic and phylogenetic total β-diversity of fish assemblages (*p* < 0.001, Figure 4a), with the taxonomic β-diversity being significantly higher (beta deviation = 0.20; *p* < 0.05, Cohen’s d = −0.64, 95% CI = [−1.11, −0.16], Figure 5a). When examining the components of decomposition, both species and phylogenetic turnover exhibited significant correlations (*p* < 0.001, Figure 4b), as well as the nestedness process (Figure 4c). Notably, taxonomic turnover (*p* < 0.05, Cohen’s d = −0.60, 95% CI = [−1.07, −0.13], Figure 5b) and nestedness patterns (*p* > 0.05, Cohen’s d = −0.11, 95% CI = [−0.58, 0.35], Figure 5c) were found to be higher than their phylogenetic equivalents. Additionally, taxonomic and phylogenetic turnover accounted for 72.23% and 67.42% of the total β-diversity, respectively (Figure 5).

### 3.4. Drivers of Fish Taxonomic and Phylogenetic Diversity in the Dongfeng Reservoir

**Figure 6:**
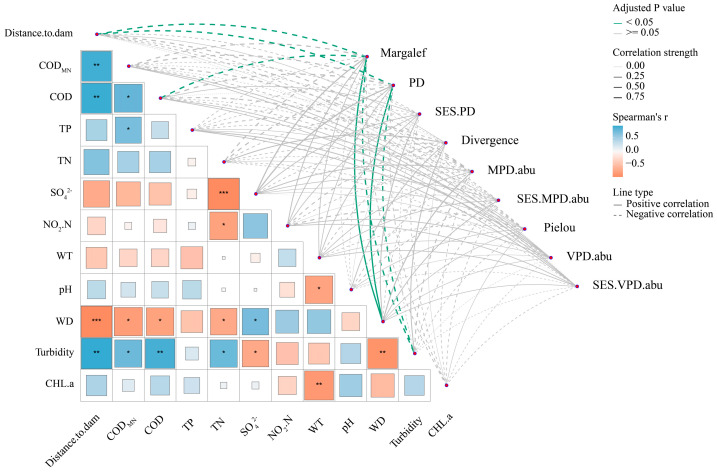
The correlation analysis between different environmental variables and taxonomic and phylogenetic α-diversity of fish assemblage in the Dongfeng Reservoir. *p* ***, *p* ** and *p* * are the significance level at 0.001, 0.01, and 0.05, respectively. Blue squares and solid green lines indicate positive correlations, while orange squares and dashed green lines indicate negative correlations.

The results showed that taxonomic (TR) and phylogenetic richness (PR) were significantly negatively correlated with various factors, including spatial distance (TR: r = −0.93, adj.*p* = 0.01; PR: r = −0.95, adj.*p* = 0.01), COD (TR: r = −0.93, adj.*p* = 0.01), and turbidity (TR: r = −0.90, adj.*p* = 0.02; PR: r = −0.87, adj.*p* = 0.03). Conversely, significant positive correlations were found for WD (TR: r = 0.88, adj.*p* = 0.03; PR: r = 0.90, adj.*p* = 0.02). No significant relationships were observed for the other phylogenetic α-diversity indices (adj.*p* > 0.05, Figure 6).

**Figure 7:**
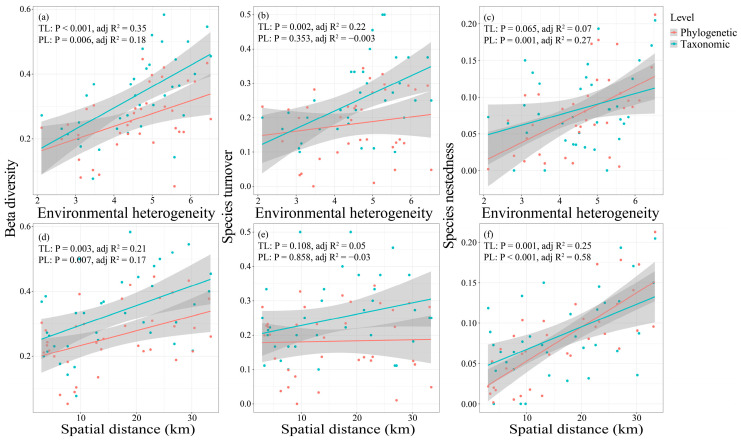
The effects of environmental heterogeneity and spatial distance on taxonomic and phylogenetic β-diversity (**a**,**d**) and its additive components ((**b**,**e**) for turnover; (**c**,**f**) for nestedness) of fish assemblages in the Dongfeng Reservoir using the distance-decay analysis. The shaded area represents the 95% confidence interval. TL and PL are taxonomic and phylogenetic level, respectively.

**Figure 8:**
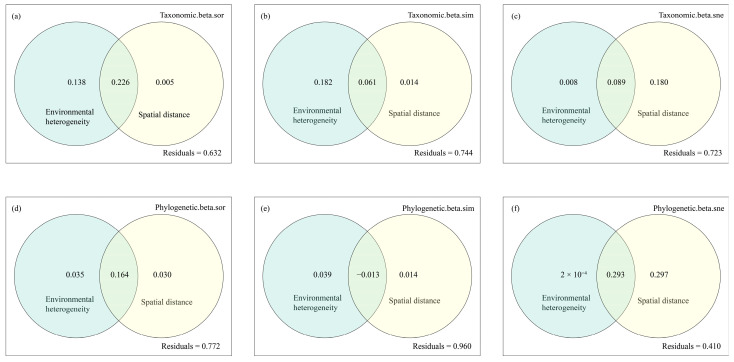
The relative importance of environmental heterogeneity and spatial distance on taxonomic and phylogenetic β-diversity and its additive components (Taxonomic level: (**a**–**c**); Phylogenetic level: (**d**–**f**)) of fish assemblages in the Dongfeng Reservoir using the hierarchical partitioning analysis.

Distance decay analysis revealed that fish taxonomic and phylogenetic β-diversity with its decomposition components increased with rising environmental heterogeneity and spatial distance (Figure 7). Specifically, total taxonomic and phylogenetic β-diversity were both significantly influenced by environmental heterogeneity and spatial distance (*p* < 0.05), with environmental heterogeneity accounting for more variance (Taxonomic Level: adj.R^2^ = 0.35; Phylogenetic Level: adj.R^2^ = 0.18) than spatial distance (TL: adj.R^2^ = 0.21; PL: adj.R^2^ = 0.17). Taxonomic turnover was significantly affected by environmental heterogeneity (*p* = 0.002, adj.R^2^ = 0.22). Taxonomic nestedness was significantly influenced solely by spatial distance, while phylogenetic nestedness was significantly affected by both environmental heterogeneity and spatial distance, with the latter explaining a greater proportion of variance (adj.R^2^ = 0.58). Additionally, hierarchical partitioning analysis revealed the similar results (Figure 8).

## 4. Discussion

### 4.1. The Homogeneous Environmental Characteristics in the Dongfeng Reservoir

Dam construction has significantly altered the hydrological characteristics of the river [34]. The results indicated that as a sampling site approaches the dam, the concentrations of COD_Mn_, COD, TN, and turbidity decrease more markedly, while SO_4_^2−^ concentration and water depth significantly increase. COD_Mn_ and COD serve as indicators of pollution from organic matter and reductive substances in the water [62]. Dam construction has reduced flow velocity in certain upstream river sections, creating a lentic or slow-flowing reservoir area where suspended particles, including sediments, organic matter, and ammonium salts, are likely to settle due to gravity. This settling primarily contributes to the observed decrease in the concentrations of these indicators in the upper layers of the reservoir. Conversely, the long hydraulic retention time in the reservoir facilitates sufficient aeration, promoting aerobic microbial decomposition of organic matter, which further explains the reduction in COD_Mn_ and COD. Additionally, on the southern side of the Dongfeng Reservoir (near site 6), a tributary named the Yangliu River, introduces substantial water that may dilute the concentrations of these indicators entering the reservoir. Under the influence of hyperpycnal flow [63], the high-velocity river water from upstream enters the reservoir, generating density flows along the reservoir bottom. This process may lead to the sedimentation of suspended solids before reaching the dam, resulting in clearer water in the upper layers in front of the dam. In terms of sulfate, the low flow velocity within the reservoir hampers the washing away of soluble substances, leading to higher concentrations compared to upstream river sections. Furthermore, Bijie City, Guizhou Province, is rich in coal resources (https://www.ccera.com.cn/web/65/202412/24703.html, accessed on 21 December 2025), and several coal mines are located upstream of the reservoir, including its tributaries. The wastewater generated from these coal mines may also contribute to the increased SO_4_^2−^ concentration in the reservoir area [64].

Previous studies have demonstrated that environmental characteristics undergo significant changes before and after dam construction, with a marked decrease in environmental heterogeneity following impoundment compared to pre-construction conditions [38]. In contrast, this study examined variations in environmental features along the gradient of the distance from the dam, revealing only a slight reduction in environmental heterogeneity within deep-water zones (Figure S3), suggesting dam impoundment contributes to environmental homogenization. Additionally, differences in methods may result in an underestimation of the response of environmental heterogeneity to damming.

### 4.2. The Significant Differences in Fish Taxonomic and Phylogenetic β-Diversity with High Turnover Pattern

The results indicated that fish community composition is significantly correlated solely with taxonomic and phylogenetic richness, suggesting that the evolutionary history length of community species increases in tandem with community richness. For instance, the fish richness (12) and phylogenetic diversity (PD, 5.23) at site 4 in this study are lower than the richness (14) and PD (6.43) observed at site 1. This phenomenon has been frequently documented in previous research [21,65]. The findings imply that, at least within this study area, phylogenetic redundancy has not occurred, or has occurred to a lesser extent, indicating that most species within the community are closely related [66]. This further suggests that as the adverse effects of dam construction on fish intensify, the community’s stability and resilience diminish. Regarding evenness and divergence, there is no significant relationship between taxonomic and phylogenetic diversity. This could be attributed to the selection of indicators that, while representing similar concepts, are fundamentally different; taxonomic and phylogenetic indices are not equivalent. For example, the abundance-based VPD index reveals changes in phylogenetic distance that are weighted by species abundance within the community, highlighting the phylogenetic differences among taxa, whereas the Pielou evenness only accounts for variations in relative species abundance. Moreover, this phenomenon might also be caused by the statistical limitations of the small sample size in this study. In future research, we will expand the spatiotemporal scale to increase the sample size and adopt more flexible sampling methods, such as stratified sampling. Notably, when the terminal species of the phylogenetic tree are randomized, a significant nonlinear relationship was found exclusively between taxonomic and phylogenetic divergence. This seemingly paradoxical result underscores the necessity of examining the relationship between taxonomic diversity and PD from multiple perspectives to deepen our understanding of the complex impacts of dam construction on community organisms.

The habitat characteristics altered by human activities tend to exhibit greater similarity, resulting in a high degree of convergence across various dimensions of community biodiversity [67,68]. The findings support the previously stated hypothesis that the taxonomic (βtax) and phylogenetic β-diversity (βphy) of fish assemblages with its additive components are significantly correlated. This correlation indicates that, at least in the context of this research area, the βphy serves as a reliable surrogate for βtax. Importantly, the βphy discussed herein primarily pertains to alterations in the terminal branches of the phylogenetic tree and excludes considerations of basal-weighted levels (i.e., turnover changes at the root and more distant nodes of the phylogenetic tree, such as the mean pairwise distance for phylogenetic information among species between communities) [69]. Existing literature indicates that the spatial patterns of tip- and basal-weighted βphy in North American freshwater fish communities exhibit significant inconsistencies [25]. Therefore, the observed strong correlation between βtax and βphy may be unidimensional, influenced by the measurement methodology of βphy. Additionally, the results revealed a positive beta deviation value (i.e., the high βtax), suggesting that these communities include a substantial number of phylogenetically related species unique to particular communities. This reflects that the reservoir has a more pronounced effect on the community composition of fish than on their evolutionary relationships. Even so, the values for βtax (0.33) and βphy (0.26) are relatively low, indicating that dam construction contributes to the homogenization of fish communities. Biological homogenization arises from complex interactions among various processes, such as shared species invasions across different communities and the extinction of endemic species [70], resulting in adverse effects on ecological, evolutionary, and social levels [71]. *C. zillii* and *L. cyanellus*, invasive fish species identified in this survey, are found at all sampling sites and may have significantly contributed to the increase in multidimensional community similarity among communities. It is anticipated that as the duration of water storage in the dam increases, the phenomenon of biological homogenization between communities will exacerbate, particularly in light of the impacts of cascade dams.

Previous research has demonstrated that, when accounting for multiple confounding factors, such as ecosystem types, geographic distance, and dispersal ability, species turnover played a significant role in contributing to overall β-diversity [31]. This study corroborates this widespread phenomenon, revealing that taxonomic and phylogenetic turnover account for 72.23% and 67.42% of the variance, respectively. The contributions of species turnover and nestedness processes to total β-diversity are contingent upon the interplay between dispersal limitation and environmental heterogeneity [15]. In homogeneous environments, high dispersal limitation increases community dissimilarity, which is primarily driven by species turnover patterns. The predominant factors influencing fish movement likely include local aquatic environmental characteristics, habitat complexity, and food availability [72], which may lead to random dispersal for certain species.

### 4.3. Environmental Heterogeneity Mainly Drives Taxonomic and Phylogenetic β-Diversity and Turnover Processes, While Spatial Distance Dominates Nestedness Processes

Identifying the drivers that influence community structure and diversity aids in the exploration of key variables and the formulation of effective management strategies [37]. Correlation analysis revealed that taxonomic and phylogenetic richness exhibited significant negative correlations with distance from the dam location, COD, and turbidity, while showing significant positive correlations with water depth. This finding further validates the previously documented nonlinear relationships between these environmental variables and distance from the dam location (Figure 2). Prior studies have established the importance of these environmental factors; for instance, turbidity affects fish predation efficiency and reproductive selection [73], while deeper water zones enhance fish abundance and alpha diversity by expanding fish spawning habitat ranges [12]. Thus, it is evident that dam construction modifies environmental characteristics in upstream reservoirs, exerting varying degrees of direct and indirect effects on fish community diversity. However, the fitting of correlations between fish diversity and multiple environmental characteristics may introduce statistical artifacts that could overstate the influence of environmental conditions.

The results showed that fish taxonomic and phylogenetic β-diversity with turnover pattern were chiefly influenced by environmental heterogeneity, thereby contradicting the second hypothesis of this study. As previously noted, the low βtax and βphy indicate that the impoundment created by the dam has modified the original environmental characteristics of the river. This alteration selectively favors certain fish species that are adapted to lentic ecosystems, thereby enhancing the community similarity and underscoring the significance of environmental factors [27]. Given the high contribution of species and phylogenetic turnover patterns, environmental heterogeneity, which is a dominant factor in the total β-diversity of fish assemblages, also emerges as a critical driver of turnover pattern. Regarding nestedness patterns, spatial distance primarily influences these patterns at both the taxonomic and phylogenetic levels. Previous study has similarly suggested that taxonomic and functional nestedness processes in Neotropical fish communities are primarily connected to spatial factors [16]. Both the upstream sections of the Dongfeng Reservoir and its tributaries contain natural river segments. As actively dispersing organisms, fish migrate between the reservoir and the rivers for feeding and predator avoidance, potentially forming nested relationships at both species and phylogenetic levels. This phenomenon indirectly reflects the heightened vulnerability of small-scale areas to mass effects [28]. Given the high connectivity among sites, random processes (e.g., ecological drift) may contribute to intricate spatial patterns [74]. It is important to note that the results of the hierarchical partitioning model exhibited a significant proportion of unexplained residuals, ranging from 41.0% to 96.0%. This phenomenon can be primarily attributed to the insufficient modeling of critical environmental factors and spatial structures [75]. Moreover, biological interactions (e.g., competition and predation) also significantly affect the distribution and diversity patterns of community organisms [76].

### 4.4. Fish Conservation Measures for the Dongfeng Reservoir

The increasing demands of humanity have underscored the undeniable benefits of dam construction. However, this development has also resulted in the fragmentation of river habitats, altered local ecological characteristics, and affected the distribution of aquatic organisms, particularly fish. The result indicated high contributions to species and phylogenetic turnover patterns, suggesting that local managers require enhanced time and financial resources to protect fish assemblages within the reservoir area. Furthermore, lesser-impacted tributaries can provide more suitable habitats for aquatic organisms, thereby safeguarding native species that are ill-suited to slow-flowing or lentic conditions and restoring essential ecosystem functions [77]. Consequently, we recommend that, in addition to artificial stocking within the reservoir area, conservation measures be implemented in major tributaries and upstream natural river sections. These measures should include enhancing habitat connectivity and establishing nature reserves to support the life history processes of rare and economically important fish species. In future research, we suggest combining the local community (i.e., LCBD) and species to overall β diversity (i.e., SCBD) to comprehensively determine priority protection areas and key species [41].

## 5. Conclusions

To the best of our knowledge, this study represents one of the limited investigations into the effects of dam construction on both taxonomic and phylogenetic α- and β-diversity of fish assemblages in the karst basin of southwest China, as well as the underlying mechanisms of community assembly. The main conclusions are as follows: (1) the taxonomic and phylogenetic α-diversity of fish assemblages in the reservoir do not always correlate, suggesting that changes in one dimension of diversity within a community cannot substitute for changes in another dimension. (2) Taxonomic β-diversity was significantly greater than phylogenetic β-diversity and both were primarily influenced by turnover processes, underscoring the importance of dispersal limitations or species dispersal capacity in homogeneous environments. (3) The patterns of taxonomic and phylogenetic β-diversity and turnover in fish assemblages were mainly shaped by environmental heterogeneity, while nestedness processes was predominantly driven by spatial distance. This represented that the formation mechanisms of fish assemblages arose from the interplay of deterministic and stochastic processes.

## 6. Recommendations

Due to the local government’s ban on fishing at the Dongfeng Reservoir, our field investigation work has been restricted in terms of time and space scales. Despite this, we believe that our results are of certain significance for understanding how the environmental characteristics and the multi-dimensional diversity patterns of fish assemblages change in lentic ecosystems. Given the absence of temporal data before the dam construction, future research should utilize “space-for-time” approaches [78,79] to facilitate an understanding of the responses in the structure and diversity patterns of aquatic communities to reservoirs, along with their underlying drivers. Moreover, taking into account the cumulative effects of cascade dams [80], we also should investigate the more extensive spatiotemporal (e.g., variations along the longitudinal gradient upstream and downstream among different seasons) effects of multiple dams on aquatic community organisms.

## Figures and Tables

**Table 1:** Fish composition in the Dongfeng Reservoir. *OF*, *RA*, *RB*, and *IRI* are occurrence frequency, relative abundance and biomass, and index of relative importance, respectively.

Order/Family/Species	*OF*	*RA*	*RB*	*IRI*
Cypriniformes				
Xenocyprididae				
*Opsariichthys bidens*	88.89	2.80	2.43	464.75
*Hemiculter leucisculus*	100	6.01	2.63	863.69
*Culter alburnus*	11.11	0.08	0.10	2.01
*Hypophthalmichthys molitrix*	11.11	0.08	9.48	106.27
Gobionidae				
*Pseudorasbora parva*	55.56	0.58	0.05	34.84
*Abbottina rivularis*	22.22	0.16	0.02	4.01
Acheilognathidae				
*Rhodeus sinensis*	66.67	3.21	0.27	231.82
*Rhodeus ocellatus*	11.11	0.49	0.08	6.36
Cyprinidae				
*Spinibarbus sinensis*	22.22	0.49	9.20	215.35
*Onychostoma yunnanense*	11.11	0.08	0.22	3.34
*Onychostoma simum*	22.22	0.33	0.27	13.24
*Cyprinus carpio*	77.78	1.07	1.09	168.02
*Carassius auratus*	100	22.55	14.69	3724.24
*Pseudogyrinocheilus prochilus*	55.56	4.77	8.22	721.86
*Discogobio yunnanensis*	33.33	0.25	0.06	10.14
Cobitidae				
*Misgurnus dabryanus*	11.11	0.33	0.08	4.60
Siluriformes				
Siluridae				
*Silurus asotus*	44.44	0.33	3.77	182.36
*Silurus meridionalis*	11.11	0.08	0.07	1.65
Bagridae				
*Tachysurus sinensis*	33.33	1.07	0.94	67.05
*Tachysurus crassilabris*	55.56	1.81	1.40	178.35
Gobiiformes				
Gobiidae				
*Rhinogobius similis*	66.67	1.15	0.03	78.83
Cichliformes				
Cichlidae				
*Coptodon zillii*	100	19.34	17.77	3711.27
Centrarchiformes				
Centrarchidae				
*Lepomis cyanellus*	100	32.92	27.14	6006.23

## Data Availability

The data presented in this study are available on request from the corresponding author.

## Supplementary Materials

The following supporting information can be downloaded at: https://www.mdpi.com/article/10.3390/ani16010145/s1, Figure S1: Individual-based species rarefaction curves for fish in the Dongfeng reservoir; Figure S2: Phylogenetic tree of fish in the Dongfeng reservoir; Table S1: The GenBank codes for fish species. Figure S3: The relationship between environmental heterogeneity and the distance from the dam.

## Abbreviations

The following abbreviations are used in this manuscript:

βtax	Taxonomic β-diversity
PD	Faith’s phylogenetic diversity
MPD.abu	Abundance-based mean pairwise distances
VPD.abu	Abundance-based variation of pairwise distances
SES	Standardized effect size
Obs.phy.alpha	Observed phylogenetic α-diversity
Mean.rand.phy.alpha	Mean of the randomized phylogenetic α-diversity
SD.rand.phy.alpha	Standard deviation of the randomized phylogenetic α-diversity
beta.dev	Beta deviation
βphy	Phylogenetic β-diversity
*IRI*	Relative importance index
F*i*	Occurrence frequency
N*i*	Proportion of individuals
W*i*	Proportion of biomass
GAMs	Generalized additive models
