# Supplementary material for: Changes in Fish Taxonomic and Phylogenetic Diversity and Their Driving Factors in a Reservoir in the Karst Basin of Southwest China
Source: Animals (Basel). 2026 Jan 5;16(1):145. doi: 10.3390/ani16010145 (PMC12784897; doi:10.3390/ani16010145)
Supplement: Supplementary file 1 [file animals-16-00145-s001.zip › animals-3965480-supplementary.pdf]

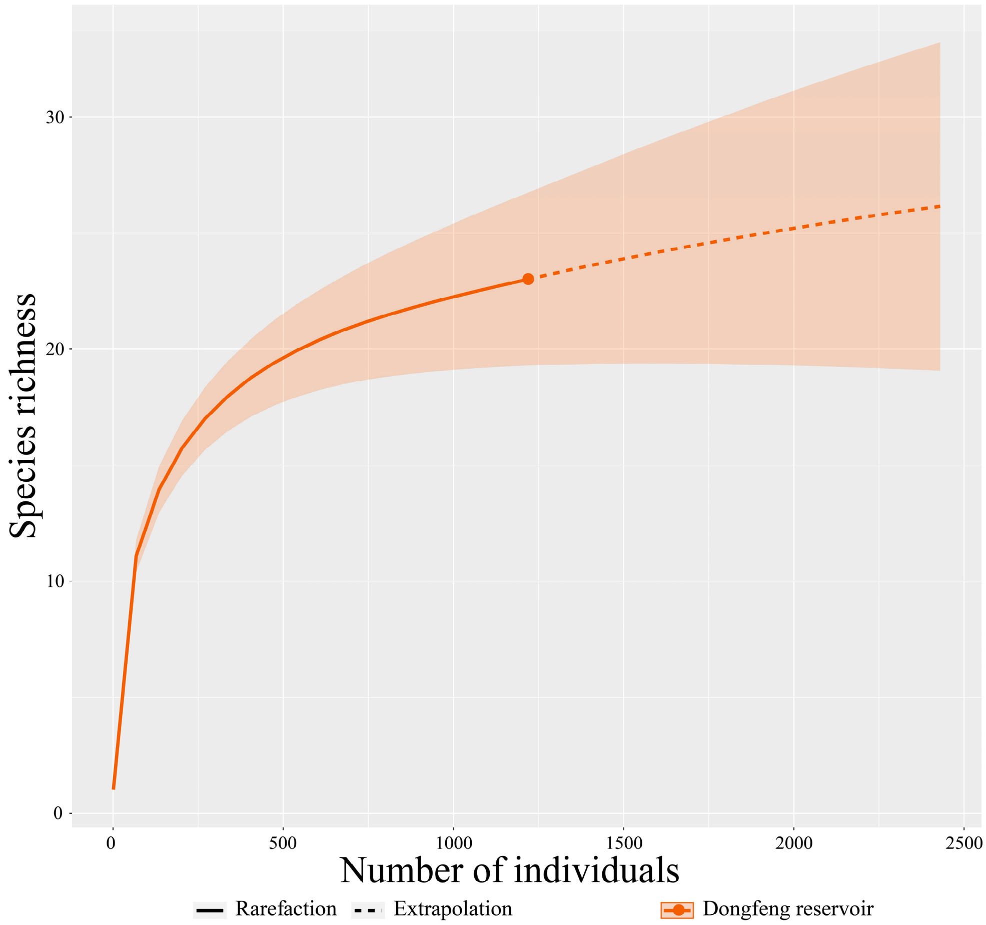

Figure S1. Individual-based species rarefaction curves for fish in the Dongfeng reservoir. Solid lines denote interpolated records of species richness, while dotted lines indicate interpolated species richness estimates with shaded 95% confidence intervals.

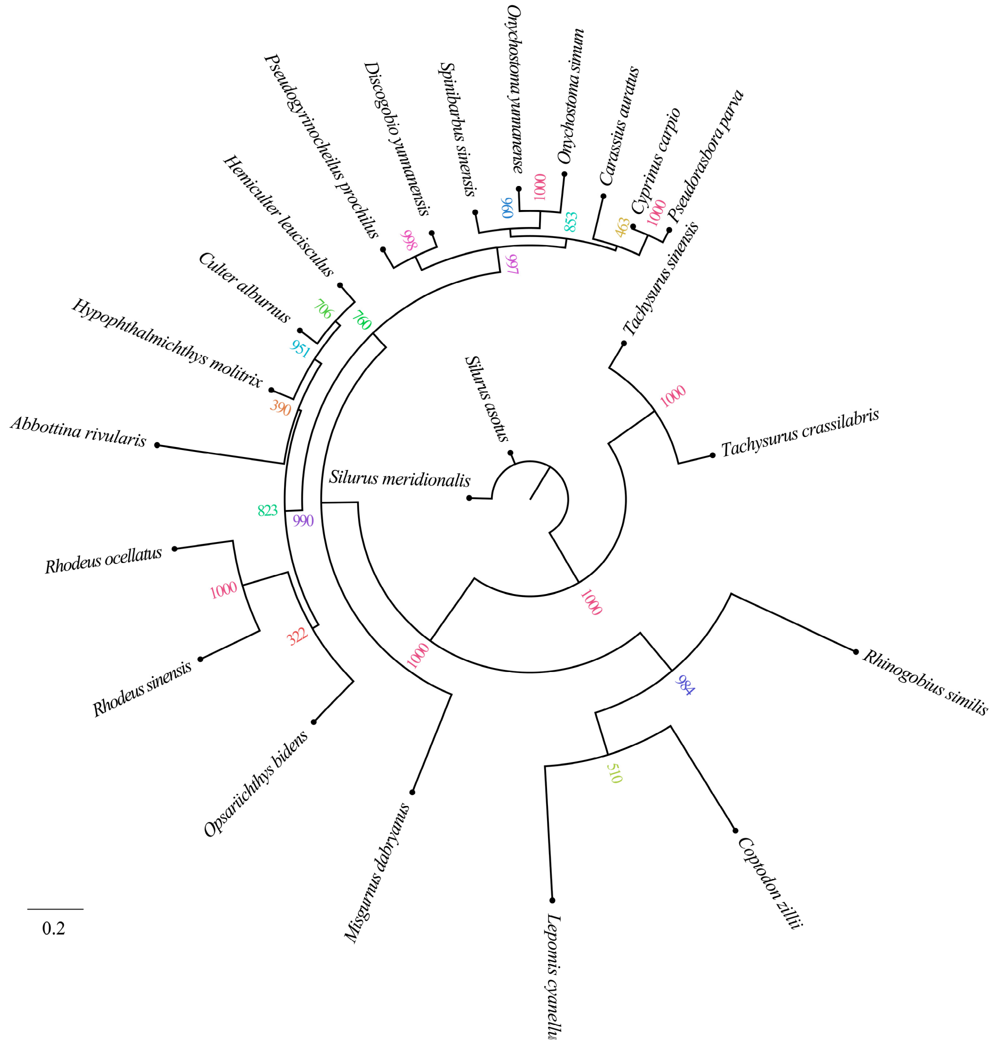

Figure S2. Phylogenetic tree of fish in the Dongfeng reservoir. The line segments and values indicate branch length and bootstrap value, respectively.

Table S1. The GenBank codes for fish species.

Species	GenBank	Species	GenBank
Opsariichthys bidens	MN832737.1	*Carassius auratus*	KF147851.1
Hemiculter leucisculus	KF956522.1	*Pseudogyrinocheilus prochilus*	KJ684987.1
Culter alburnus	GU190362.1	*Discogobio yunnanensis*	KJ997760.1
Hypophthalmichthys molitrix	MW344880.1	*Misgurnus dabryanus*	KJ027397.1
Pseudorasbora parva	KJ135626.1	*Silurus asotus*	MK895951.1
Abbottina rivularis	KJ183133.1	*Silurus meridionalis*	HQ907992.1
Rhodeus sinensis	KF533721.1	*Tachysurus sinensis*	NC015888.1
Rhodeus ocellatus	KT004415.1	*Tachysurus crassilabris*	JX867257.1
Spinibarbus sinensis	KC579368.1	*Rhinogobius similis*	KF371534.1
Onychostoma yunnanense	MN395748.1	*Coptodon zillii*	KM658974.1
Onychostoma simum	KF021233.1	*Lepomis cyanellus*	KC427094.1
Cyprinus carpio	OL699932.1		

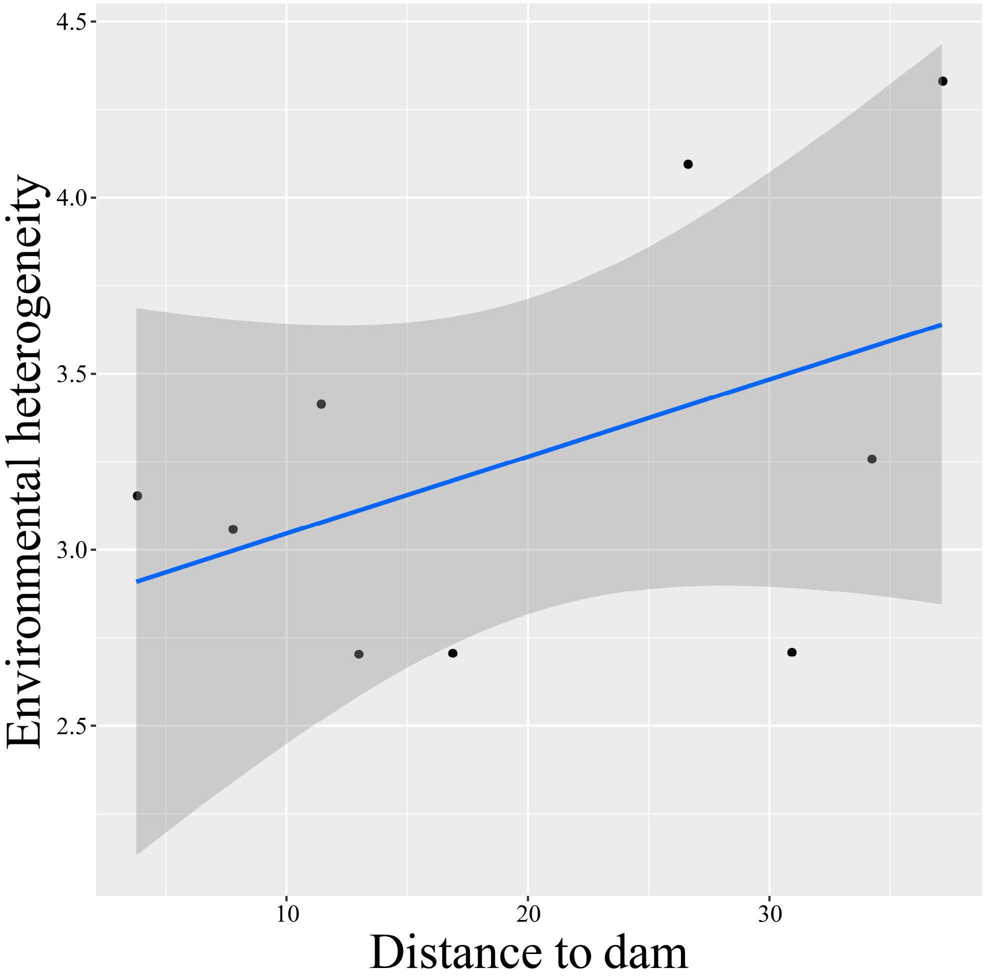

Figure S3. The relationship between environmental heterogeneity and the distance from the dam. The solid blue line represents the fitting curve with shaded 95% confidence intervals.
